# Assessing the Predictive Capabilities of Autoregressive Integrated Moving Average and Linear Regression Models for Acute Changes in Clinical and Selected Laboratory Parameters in Children After Cardiac Surgery in the ICU

**DOI:** 10.3390/children11111312

**Published:** 2024-10-29

**Authors:** Sharmin Nahar Sharwardy, Hasan Sarwar, Mohammad Nurul Akhtar Hasan, Mohammad Zahidur Rahman

**Affiliations:** 1 Department of Computer Science and Engineering, Jahangirnagar University, Savar 1342, Bangladesh; rmzahid@juniv.edu; 2 Department of Computer Science and Engineering, United International University, Dhaka 1212, Bangladesh; hsarwar@cse.uiu.ac.bd; 3 Department of Pediatric Cardiac Intensive Care, National Heart Foundation Hospital and Research Institute, Dhaka 1216, Bangladesh; drhasan.pediicu@gmail.com

**Keywords:** congenital heart diseases, pediatric, linear model, ARIMA model, cause and effect, ICU

## Abstract

(1) Background: The main objective of this research was to assess the clinical factors related to the condition of pediatric patients with congenital heart defects after they underwent intensive care unit surgery. The information was gathered from the Congenital Heart Disease Surgery Unit at the National Heart Foundation Hospital and Research Institute in Dhaka, Bangladesh. We gathered and examined data from 288 ICU patients. Patients under the age of twelve who required more than a 24-h ICU stay were selected. (2) Methods: The dependent and independent variables were chosen in advance based on expert opinion. The relationships between these pre-specified ICU parameters were determined using the Pearson correlation model and assessed through linear regression and ARIMA modeling to predict subsequent acute changes in the patients’ ICU statuses. (3) Results: A statistically significant relationship (*p* value < 0.001) was found between CVP and BP (95% CI = 0.2113; 0.353 r = 0.2841249) and between PEEP and FiO_2_ (95% CI = 0.6992; 0.770 r = 0.7367744). Although the relationships between pH and PO_2_ were minor (95% CI = 0.161; 0.308 r = 0.2362575), they were statistically significant. The parameters considered statistically significant (*p* < 0.001) were chosen for forecasting. In this work, the linear regression model and the ARIMA model used the parameters BP, FiO_2_, and PO_2_ for prediction. We forecasted the patients’ statuses for the next hour. It was found that the ARIMA model had a lower error rate than the linear regression model. (4) Conclusions: This study helps identify the important parameters for predicting and monitoring patients’ statuses in the ICU, with the ultimate goal of providing physicians with an early warning system to anticipate deterioration in clinical and biochemical parameters. The ability to accurately forecast future patients’ conditions can enable proactive, targeted interventions, potentially improving outcomes and reducing the risk of adverse events.

## 1. Introduction

Pediatric ICU patients generally fall within the age group of 0–18 years, with complications mostly arising from congenital heart diseases (CHDs) from birth. Recently, we have seen a rise in neonatal admissions to pediatric cardiac ICUs. An Indian study showed a 30–50% increase in the admission of child patients due to cardiac diseases [1]. Another study showed that 32,000 children in the USA are affected by CHDs, of which 25% require invasive treatment [2,3]. Birth defect-associated CHDs contribute to a 30–40% mortality rate among infants and children [4]. Although several advanced interventional and surgical techniques have been devised in recent years, heart disease in infants and children remains a major cause of morbidity and mortality [5]. Winter admissions further increase the number of pediatric patients per unit and the associated mortality rates [6]. Moreover, the number of pediatric ICUs is not adequate globally, and most low- and middle-income countries do not have designated ICUs with adequately trained staff, characterized by a critically low nurse-to-patient ratio [7].

Predicting ICU patients’ statuses in terms of immediate changes, especially the deterioration in some clinical and biochemical parameters, may improve the quality of treatment by allowing doctors to be more proactive in anticipating patient deterioration and helping physicians prepare families for likely changes in their child’s clinical status. Timely intervention may also reduce the length of hospital/ICU stays, which could reduce patient costs and free up more hospital beds to accommodate other patients in need.

Several studies have employed time-series modeling for sensing or predicting the clinical status of patients. Kennedy and Turley [8] outlined the processes required to construct predictive models employing time-series clinical data, as well as prediction models from nonclinical domains that use time-series data. The authors demonstrated the methodology with the specific example of developing a prediction model for cardiac arrest in a pediatric intensive care unit. Muge Capan et al. [9] predicted the hospital census for neonatal ICU patients. They showed that time-series modeling could predict the census with fewer errors compared to the fixed-average census approach, although the inputs were static variables. Yixian Xu et al. [10] proposed a model for predicting ICU mortality in rheumatic heart disease. A framework based on machine learning was published to forecast cardiac arrests in a pediatric intensive care unit [11]. To assess predictability, the authors leveraged causally related changes in signals such as heart rate, respiration rate, systolic blood pressure, and peripheral cutaneous oxygen saturation. Meanwhile, M. Pishgar et al. [12] predicted unplanned 30-day readmissions of ICU patients with heart failure. Demographic information and severity scores were used for the predictions. Some papers have analyzed the early prediction of critical events for infants. A machine learning model was employed by Bongjin Lee et al. [13] to forecast fatal outcomes during the initial stages of intensive care unit admission. Victor M. Ruiz et al. [14] focused on the early prediction of critical events for infants. None of these methods, however, offers a comprehensive framework for expertly enhanced causal graph construction with a focused clinical application. However, the identification of dominant parameters for predicting upcoming clinical and/or biochemical deterioration may help decrease the mortality rate.

With the ultimate goal of giving doctors an early warning of a patient’s immediate future condition during ICU stays and enabling better healthcare services by enhancing patient information, this study aims to identify the critical parameters measured in intensive care units to monitor the status of patients and predict the future values of those parameters. Here, status refers to the prediction of ICU patients’ conditions in terms of immediate changes, especially the deterioration of some clinical and biochemical parameters, which will guide caregivers in taking appropriate and adequate management strategies to avoid anticipated clinical adversities.

## 2. Materials and Methods

### 2.1. Data Collection and Preprocessing

Data on 300 patients were gathered from the hospital. Patients were admitted to the ICU at the National Heart Foundation Hospital and Research Institute in Dhaka, Bangladesh, between 2017 and 2018. Data from patients who stayed more than 24 h in the ICU were collected for this study. This study included patients with congenital cardiac disease who were between the ages of one day and twelve years. So, patients older than 12 years were excluded from this study, resulting in 12 patients being excluded. So, data on 288 patients were used for this analysis. Among them, 146 (51%) were female patients, and 142 (49%) were male patients. In this dataset, 3 (1 %) were neonates (<1 month), 35 (12%) were infants (<1 year), 74 (26%) were toddlers (1 to 3 years), and 176 (61%) were older children (≤12 years).

According to most definitions, individuals up to 18 years are considered children. Patients older than 12 years were excluded as their congenital heart disease and associated conditions begin to resemble that of adult congenital heart disease patients, which include several longer-term comorbidities and physiological changes that are not seen as frequently in younger patients.

**Figure 1:**
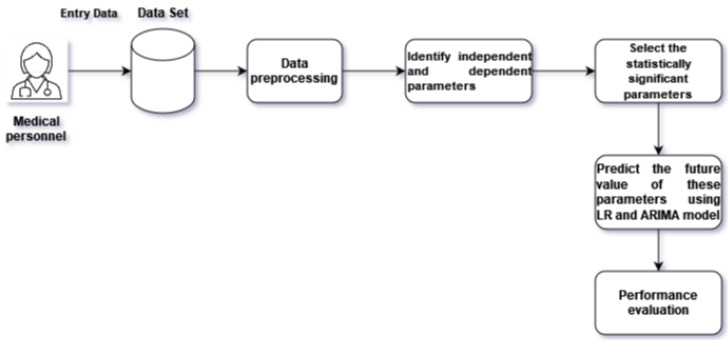
Workflow diagram.

The patients were always under observation by medical personnel, including doctors and nurses, in the intensive care unit. The medical personnel held a piece of paper listing 42 parameters. They tracked these 42 parameters while observing the patients. Each patient’s data were gathered every hour. A typical patient remained in the ICU for two to seven days. Each day, a large datasheet was created. The collected patient variables included demographic information like age, sex, weight, date of ICU admission, and total length of ICU stay. The physiological data recorded in this dataset included arterial blood pressure (ABP), central venous pressure (CVP), body and peripheral temperature, and peripheral capillary oxygen saturation (SpO_2_). Ventilation data included in this study were the mode of ventilation, ventilation rate, tidal volume (TV), peak inspiratory pressure (PIP), positive end-expiratory pressure (PEEP), mean airway pressure (MAP), fraction of inspired oxygen (FiO_2_), and arterial and venous blood gas measurements (pH, pCO_2_, and bicarbonate). Laboratory findings included levels of calcium, potassium, chloride, sodium, urine, and anion gap. The primary drugs included in our dataset were Dobutamine, Norepinephrine, Milrinone, Amiodarone, and Furosemide. The dataset contained many inaccuracies because of noise, missing values, or incorrect records. As a result, we needed to recognize and address these conflicting or inaccurate records. First, we noticed that the measurement units for some variables varied; for instance, the peripheral temperature was expressed in Celsius. Second, some parameter values were recorded as errors or were missing entirely. Third, some variables had multiple recorded values at the same time. We performed forward and backward imputation for data occasionally missing within a 24 h period. If there were no such data for 24 h, we used the average values of that variable from all patients. For instances with multiple values recorded in an hour, we randomly selected one value. We represented all data in Fahrenheit to solve the problem of conflicting values in body temperature records. In this paper, we provide an analysis of the cause-and-effect relationships between ICU parameters. The Pearson correlation model was used to find statistically significant correlations. Then, we predicted patients’ statuses using the ARIMA model and the simple linear regression (LR) model and analyzed the differences between the two models. Figure 1 shows the workflow diagram of this study.

This study’s protocols and procedures followed the ethical rules and principles of the Declaration of Helsinki. Ethical approval was obtained from the National Heart Foundation Hospital and Research Institute Ethics Committee (Decision No. 4/14-7/AD-2230/2022).

### 2.2. Cause-And-Effect Relationship

Cause-and-effect statements demonstrate a distinct, direct connection between events. They demonstrate how one action or occurrence triggers another [15]. By working with a clinical domain expert, a critical care physician, we created a causal graph using our knowledge of the clinical domain. Three operations are involved in the causal relationship: if the endpoint is supported by domain knowledge, preserve it; if not, modify the direction or orientation of the endpoint if the domain information supports it; and if neither applies, remove the endpoint. Statistical analyses were performed using the R programming language. The Pearson correlation test was used to conduct the correlation analysis. The threshold for statistical significance was set at *p* < 0.01.

### 2.3. Linear Model and ARIMA Model

A predictive, quantifiable technique for showing the relationship between a dependent variable and a specific configuration of independent variables is called linear regression. It is an easy method for showing how one dependent variable and one or more independent variables are related. It is referred to as simple linear regression when there is just one independent variable present. The process is known as multiple linear regression when there are numerous independent variables involved. Linear regression was used in this study to forecast patients’ statuses [16].

The linear equation that adds specific information, denoted as arrangement x, to the linear regression description is described as follows: the response y is the predicted outcome for the data given a particular arrangement (*y*). Each value is provided by the linear equation or a portion of a scale factor known as the coefficient (represented by the Greek letter beta β). Adding more coefficients in the same manner gives the equation more degrees of freedom, and one of these coefficients is frequently referred to as the intercept or offset coefficient. For a simple regression problem, the model is as follows:

(1) $$Y_{i}=\beta_{0}+\beta_{1}x$$

where $\beta_{0}$ is the intercept, $\beta_{1}$ is the coefficient, *x* is the independent variable, and *y* is the dependent variable [16].

Autoregressive integrated moving averages are limited to being linear functions of prior data, exhibiting linearity in their projections of future values [17]. ARIMA, which stands for autoregressive integrated moving average, is a statistical technique used to analyze time-series data. The three main components of an ARIMA model are an autoregressive (AR) process, an integrated (I) process, and a moving average (MA) process. The ARIMA model, sometimes known as the Box–Jenkins model [18], is the most popular method for predicting future values in time-series forecasting. According to [19], Equation 5 is used to compute the future value of a variable by summing the values from the past.

(2) $$X_{t}=b_{0}+a_{1}X_{t-1}+\cdots +a_{p}X_{t-p}+e_{t}-b_{1}e_{t-1}+\cdots +b_{q}e_{t-q}$$

In this case, the error is denoted by e subscript *t*, the number of moving averages is denoted by *q*, the number of autoregressive terms is denoted by *p*, and the real value at time *t* is represented by *X* subscript *t*.

The mean absolute error (MAE) and the root mean square error (RMSE) were used as indices to evaluate the performance of the model. The MAE measures the absolute error compared to the actual and expected values. The root mean square error (RMSE) is the square root of the average of the squared differences between the actual and anticipated values. These functions are based on the following equations [20]:

(3) $$MAE=\sum \frac{1}{N}\left|y-\hat{y}\right|$$

(4) $$RMSE=\sqrt{\frac{1}{N}\sum {\left(y-\hat{y}\right)}^{2}}$$

The *MAE* and *RMSE* are represented by Equations (3) and (4), respectively. The real value in this example is *y*, while the predicted value is $\hat{y}$.

## 3. Results

**Figure 2:**
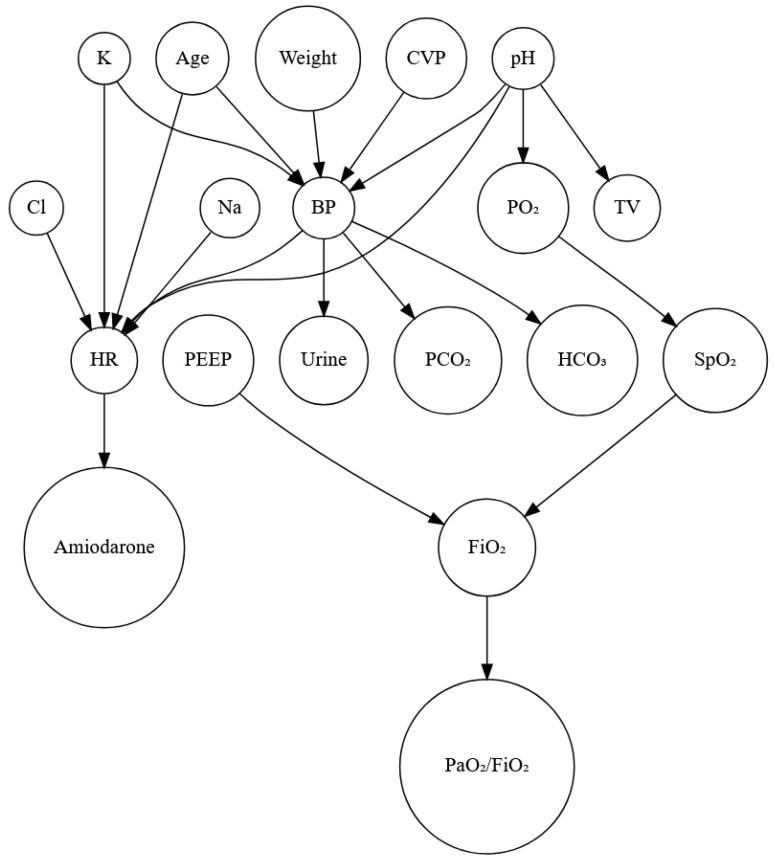
Causal graph after expert evaluations were utilized to include domain knowledge. Amiodarone is a medication that prevents and treats an irregular heartbeat.

This study included 288 post-operative pediatric patients with CHD. In health studies, variables typically fall into two categories. We anticipated that the independent variables ($Node_{from}$) would impact the dependent variables ($Node_{to}$) (predictors). What occurs as a consequence of an independent variable is referred to as a dependent variable or predictor. The dependent and independent variables were selected according to the designated expert’s advice. The procedure for using the advice of the domain expert is shown in Table 1. $Node_{from}$ to $Node_{to}$ means $Node_{from}$ causes $Node_{to}$. After consulting a physician, we evaluated the graph to ensure it was acyclic. Figure 2 shows the cause-and-effect relationship between the parameters.

**Figure 3:**
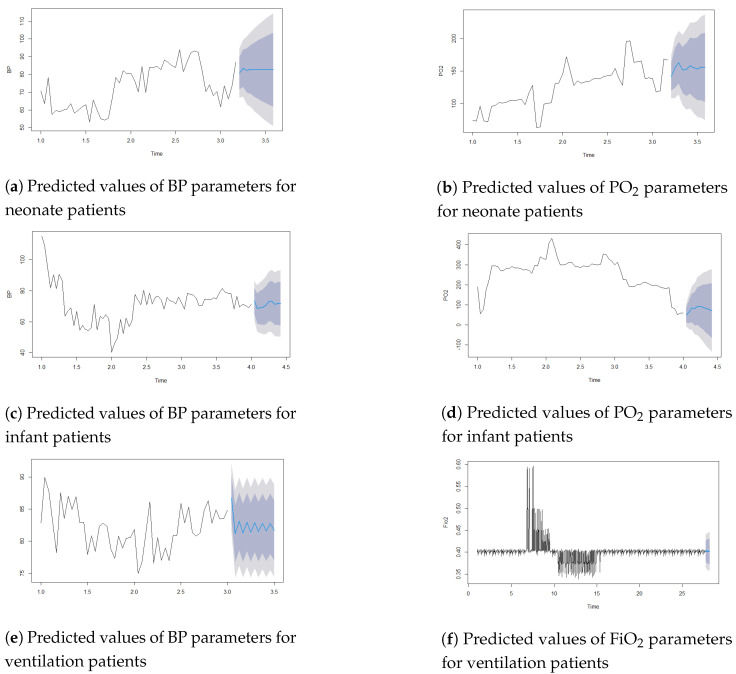
Forecasting patients’ conditions for the next hour using the ARIMA model.

**Figure 4:**
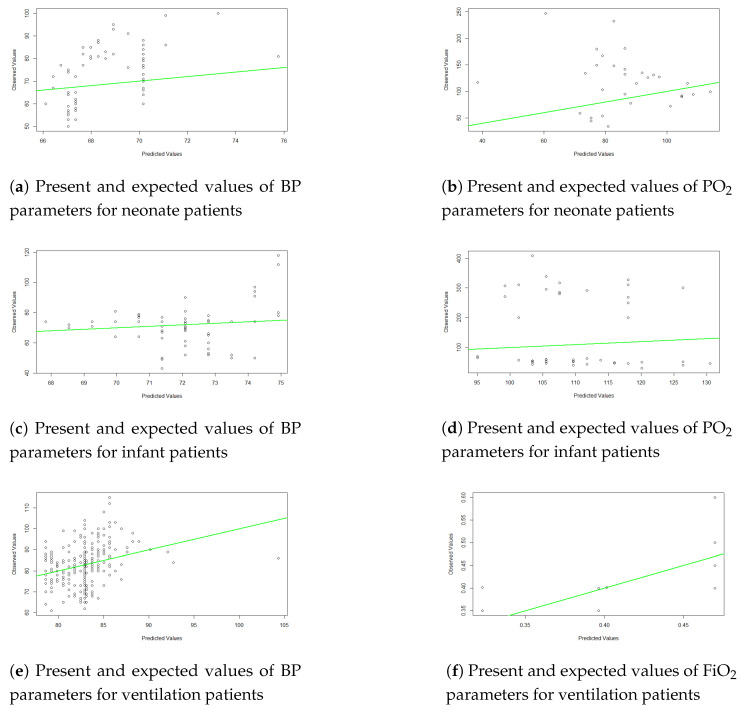
Forecasting patients’ conditions for the next hour using the linear regression model.

According to expert knowledge, weight and BP are confounding variables that can influence other independent and dependent variables. So, we eliminated these weight–BP correlations. Table 2 demonstrates that BP and urine output were significant (r = 0.151079; *p* < 0.001), pH and PO_2_ were significant (r = 0.2362; *p* < 0.001), and PEEP and FiO_2_ had a high correlation (r = 0.7367744; *p* < 0.001) and were significant. So, for forecasting patients’ statuses, this study used three parameters: BP (mmHg), PO_2_ (mmHg), and FiO_2_(%). To predict patients’ statuses, the LR and ARIMA models were used. Figure 3 shows the predicted values of the BP, PO_2_, and FiO_2_ parameters for the next 10 h using the ARIMA model. Here, the blue line indicates the expected value, and the upper and lower prediction limits are indicated by shaded areas. Figure 4 shows the actual and predicted values using the LR model.

For the ARIMA model, the autoarima function was used in R and R Studio version 2.1.1. For neonate patients, the best ARIMA model selected was (2, 1, 1); for infant patients, the best ARIMA model selected was (1, 1, 0); and for ventilation patients, the best ARIMA model selected was (1, 0, 2). This study used the first 48 h of time-series data for prediction, utilizing a univariate time-series dataset for analysis. The lowest errors in forecast accuracies are shown in Table 3. This study showed minimal errors in the MAE (BP = 2.17; ${\mathrm{FiO}}_{2}$ = 0.009) for ventilation patients. For neonate patients, the MAE = 5.2274 and the RMSE = 7.001 for the BP parameters, while the MAE = 10.83 and the RMSE = 16.04 for the ${\mathrm{PO}}_{2}$ parameters in the ARIMA model.

## 4. Discussion

Every year, CHD causes significant morbidity and mortality, particularly in neonates, despite significant advances in medical and surgical treatment. Studying the dominant parameters affecting outcomes after surgery and identifying a model that helps predict the patient’s course in the acute setting could significantly advance care.

Our study presented a cause-and-effect relationship from observational data using expert knowledge. Nowadays, the ability to determine the causal consequences of medical therapies through data analysis makes causal inference an essential topic in the medical industry [25]. However, potential outcome guidelines (Rubin causal model) [26,27] and structural causal models (SCMs) [28] are considered the foundation of causal models because they consistently incorporate prior causal knowledge, assumptions, and estimates. Using expert information from clinicians or previously published literature, structure learning algorithms must be used to create a graphical representation of the causal link between variables of interest in this approach [29]. However, a variety of algorithms exist that can determine the graphical structure from data under various presumptions. As a result, depending on the underlying assumptions, the resulting causal graph may differ significantly [30]. Although there exist additional clinical applications of SCMs [31,32], the majority of the literature mainly utilizes domain expertise to create causal graphs. This study created a casual graph using clinical expertise, as shown in Figure 1. A significant association between the parameters is shown in Table 2. Although some of the parameters were highly statistically significant, the correlations were quite low for most of the parameters. Our study showed that BP and CVP (r = 0.284), pH and ${\mathrm{PO}}_{2}$ (r = 0.23), and PEEP and ${\mathrm{FiO}}_{2}$ (r = 0.734) exhibited significant correlations. BP may affect other parameters, while ${\mathrm{PO}}_{2}$ determines the arterial blood oxygenation status, reflecting the gas exchange in the lungs. So, these parameters signal changes that require close monitoring and timely intervention.

This study also used a simple linear regression model and an ARIMA model for predicting patient conditions in the ICU. The prediction of patient outcomes in the intensive care unit (ICU) setting is a critical challenge that has garnered significant research attention. While traditional statistical models, such as linear regression and ARIMA (autoregressive integrated moving average), are often applied to this task, they often fall short of capturing the complex and nonlinear relationships inherent in ICU patient data. In this study, the ARIMA model showed fewer errors than the linear regression model.

One of the key challenges in using ARIMA models for ICU patient data is the limited availability of historical data. Typically, ARIMA models require a relatively long time series of data to accurately capture the underlying patterns and trends [33,34]. In the context of ICU patient data, the duration of the stay and the rapid changes in the patient’s condition can lead to short and irregularly spaced time series, making it difficult for ARIMA models to perform accurate forecasts [35].

The limitations of this study include the consideration of confounding parameters for cause-and-effect relationships in this study.

Preload, afterload, and contractility also play a significant role in determining cardiac output. However, these factors are rarely assessed directly in a clinical setting and are only monitored in a very small number of highly specific cases or for research purposes in locations with the necessary infrastructure and hardware. The availability of suitable technology to assess preload and afterload directly is very limited. In any case, CVP can serve as a stand-in for preload as it provides sufficient and useful information. However, there is an indirect relationship between afterload and blood pressure, urine output, and peripheral temperature. It is correct that an echocardiography can be used to measure functional parameters, and this is often performed. In considering this, the analysis should have incorporated it. As it was left out of the analysis, it can be considered a limitation of this study. We also acknowledge the importance of serum lactate after cardiac surgery. However, as mentioned, these values could not be used due to frequently missing records. Serum lactate values are typically obtained alongside arterial blood gas analysis, along with many other parameters. Due to sensor malfunction, the serum lactate values were frequently missing and were not included in this study. Many other factors, such as preoperative hemodynamics, nutritional status, renal dysfunction, and neurological state are also important determinants of short- and long-term outcomes after surgery. However, including these parameters could have made the analysis more complex and time-consuming; hopefully, this can be addressed in our future research. Due to limited resources, most of the surgeries were performed electively. Patients who had severe hemodynamic anomalies, renal impairment, or neurological dysfunction were not admitted for surgery.

Moreover, ICU patient data often exhibit complex and nonlinear relationships that may not be adequately captured by the linear structure of ARIMA models [36,37]. Patients’ disease risk and mortality are influenced by a multitude of factors, including clinical characteristics, demographic information, and treatment interventions, which can introduce significant heterogeneity and nonlinearity into the data [38].

To address these limitations, our future work will explore the use of more advanced machine learning techniques, which have demonstrated improved performance in predicting ICU patient outcomes.

## 5. Conclusions

The intensive care unit is a critical care setting that generates a wealth of patient data, including vital signs, laboratory results, and other clinical parameters. Effectively leveraging these data to monitor patients’ statuses and predict future outcomes can significantly improve clinical decision making and patient outcomes. This study aimed to investigate the relationship between various ICU parameters and explore the use of time-series and regression models to predict the future values of those parameters. The Pearson correlation model was employed to identify statistically significant correlations between ICU parameters. Then, the autoregressive integrated moving average model and simple linear regression model were used to predict patients’ statuses, with the goal of assessing the performance of these two approaches. The findings of this study indicate that the ARIMA model outperformed the linear regression model in terms of prediction accuracy, demonstrating lower error rates. This suggests that the ARIMA model was more predictive but still requires ongoing refinements and technology to help physicians in real time. The results of this study highlight the importance of leveraging advanced analytical techniques to monitor and predict a patient’s acute course in the ICU. By identifying the most important physiological parameters that are predictive of patient outcomes, clinicians can be provided with an early warning system to intervene and improve patient care.

## Figures and Tables

**Table 1:** Expert knowledge of casual relationships.

${\mathrm{Node}}_{\mathrm{from}}$	${\mathrm{Node}}_{\mathrm{to}}$	Evidence
Weight	BP	Domain Expert [21]
BP	HR	Domain Expert [21]
CVP	BP	Domain Expert [22]
BP	Urine output	Domain Expert [21]
Age	HR	Domain Expert
Age	BP	Domain Expert [21]
BP	${\mathrm{PCO}}_{2}$	Domain Expert
BP	${\mathrm{HCO}}_{3}$	Domain Expert
Na	HR	Domain Expert
Cl	HR	Domain Expert
PEEP	${\mathrm{FiO}}_{2}$	Domain Expert [23]
${\mathrm{SpO}}_{2}$	${\mathrm{FiO}}_{2}$	Domain Expert
${\mathrm{FiO}}_{2}$	${\mathrm{PaO}}_{2}{\mathrm{/FiO}}_{2}$	Domain Expert [24]
HR	Amiodarone	Domain Expert
K	BP	Domain Expert
K	HR	Domain Expert
PH	${\mathrm{PO}}_{2}$	Domain Expert
pH	TV	Domain Expert
pH	HR	Domain Expert
pH	BP	Domain Expert
${\mathrm{PaO}}_{2}$	${\mathrm{SpO}}_{2}$	Domain Expert [24]

BP—blood pressure; CVP—central venous pressure; HR—heart rate; ${\mathrm{pCO}}_{2}$—partial pressure of carbon dioxide; ${\mathrm{HCO}}_{3}$—bicarbonate; ${\mathrm{SpO}}_{2}$—oxygen saturation; Na—sodium; Cl—chloride; k—potassium; PEEP—positive end-expiratory pressure; ${\mathrm{FiO}}_{2}$—fraction of inspired oxygen; ${\mathrm{PaO}}_{2}$—partial pressure of oxygen; TV—tidal volume; ${\mathrm{PaO}}_{2}{\mathrm{/FiO}}_{2}$—the ratio of arterial oxygen partial pressure to fractional inspired oxygen.

**Table 2:** Relationships between independent and dependent variables.

Predictor	Independent Variable	*p* Value	Correlation	*t*-Test
HR	BP	0.7631	0.01191929	0.30156
HR	Na	0.07837	0.0695229	1.7631
HR	Cl	0.1415	0.05808523	1.4719
HR	K	0.1022	−0.06456345	−1.6368
HR	PH	0.725	−0.01390998	−0.35193
BP	CVP	<0.001 *	0.2841249	7.4968
BP	K	0.5047	−0.02637669	−0.66752
BP	pH	0.002025 *	−0.1216032	−3.0993
Urine output	BP	0.0001217 *	0.151079	3.8664
${\mathrm{PCO}}_{2}$	BP	0.7896	−0.0105502	−0.26692
${\mathrm{HCO}}_{3}$	BP	0.5382	−0.02433762	−0.61588
${\mathrm{FiO}}_{2}$	PEEP	<0.001 *	0.7367744	27.567
${\mathrm{FiO}}_{2}$	${\mathrm{SpO}}_{2}$	0.7339	0.01344123	0.34007
${\mathrm{PaO}}_{2}{\mathrm{/FiO}}_{2}$	${\mathrm{FiO}}_{2}$	0.0002411 *	−0.1444255	−3.6924
${\mathrm{PO}}_{2}$	pH	<0.001 *	0.2362575	6.151
TV	pH	0.01845	0.09298063	2.3625
${\mathrm{SpO}}_{2}$	${\mathrm{PO}}_{2}$	0.5347	0.2362575	0.62112

Here, * indicates *p* < 0.01.

**Table 3:** The performance evaluation metrics.

Model	Neonate Patients		Infant Patients		Ventilation Patients	
	MAE	RMSE	MAE	RMSE	MAE	RMSE
LR Model (BP)	5.973879	8.0574	2.795142	6.707442	4.47021	4.974626
LR Model (${\mathrm{PO}}_{2}$)	38.5670	49.01	81.72645	99.54716	-	-
LR Model (${\mathrm{FiO}}_{2}$)	-	-	-	-	0.0122449	01503418
ARIMA Model (BP)	5.2724	7.0011	4.435455	6.362786	2.176254	2.618761
ARIMA Model (${\mathrm{PO}}_{2}$)	10.8366	16.0403	16.065	27.812	-	-
ARIMA Model (${\mathrm{FiO}}_{2}$)	-	-	-	-	0.0095	0.01941237

Gray boxes indicate lowest errors. LR = linear regression model; ARIMA = Autoregressive integrated moving average.

## Data Availability

For privacy considerations, the authors will provide the raw data that support the findings of this paper upon request.
